# Co-occurrence of risk factors for non-communicable diseases among in-school adolescents in Tanzania: an example of a low-income setting of sub-Saharan Africa for adolescence health policy actions

**DOI:** 10.1186/s12889-019-7320-1

**Published:** 2019-07-22

**Authors:** Festo K. Shayo

**Affiliations:** 1 Department of Internal Medicine, Muhimbili University of Health and Allied Sciences, P. O Box 65000, Dar es Salaam, Tanzania; 2 Department of Global Health Entrepreneurship, Division of Public Health, Graduate School of Tokyo Medical and Dental University, Tokyo, Japan

**Keywords:** Co-occurrence, Risk factors, Non-communicable diseases, In-school adolescents, Tanzania, Sub-Saharan Africa

## Abstract

**Background:**

Childhood lifestyle, health-risk behaviours contribute to two-thirds of non-communicable diseases (NCDs) premature mortality in adult populations. The co-occurrence of risk factors for NCDs is more harmful to health than that of individual risk factor effects when are added independently. The main objective of the present study was to explore the prevalence, sociodemographic distribution, and the co-occurrence of risk factors for NCDs among in-school adolescents.

**Methods:**

The present study is based on the secondary analysis of the first nationwide representative sample of the 2014 Tanzania Global School-based Student Health Survey (GSHS). A total sample of 3,793 in-school adolescents was included in the present analysis. The dependent variables were as follows: an unhealthy diet, physical inactivity, tobacco use, excessive alcohol use, and suicide attempt. The analysis involved the Chi squire χ^2^ test, multinomial and multivariate regression models: to determine the association between the variables of interest. In all analyses, the set level of statistical significance was a *p*-value of less than 0.05 at 95% confidence intervals.

**Results:**

The most prevalent combination of risk factors for NCDs were as follows: unhealthy diet and physical inactivity 666 (17.6%); unhealthy diet and suicide attempt 151 (4.0); unhealthy diet and tobacco use 98 (2.8); and unhealthy diet, physical inactivity, and suicide attempt 81 (2.1). In the adjusted regression model; having three 0.60 [0.40–0.91], and a sum of four and five 0.46 [0.28–0.79] risk factors than having no risk factor showed a significant declined with increasing in adolescents age. Primary in-school adolescents than secondary in-school adolescents were significantly more likely to have two 1.81 [1.42–2.32], three 2.40 [1.63–3.54]; and a sum of four and five 2.90 [1.61–5.13] combinations of risk factors.

**Conclusion:**

The co-occurrence of lifestyle health-risk factors for NCDs was prevalent among in-school adolescents: it was significantly higher among younger adolescents. A multi-strategy public health intervention program may be more effective than that of a single risk factor approach: therefore, suitable for resource-limited settings, such as Tanzania.

## Background

Globally, NCDs are the leading cause of premature mortality and morbidity, however, they are potentially preventable [1]. The World Health Organization (WHO) identified four main NCDs as a leading cause of mortality: Cardiovascular disease (CVD), Diabetes mellitus, Chronic respiratory diseases, and Cancers. These four diseases share the four potential modifiable risk factors NCDs: physical inactivity, unhealthy diet, harmful alcohol use, and tobacco use [1]. Studies showed that the modifiable risk factors are usually established during adolescence and are then carried to adulthood [2, 3]. The four modifiable risk factors contribute to over 80% of NCDs premature mortality [1]. Up to 80% of CVD, type 2 diabetes mellitus, and over one-third of cancers can be prevented by reducing or eliminating the four modifiable shared risk factors [4].

On the other hand, mental health disorders have an inextricable association with NCDs and their outcomes [5]. There is increasing in knowledge about the complexity and bidirectional causality between mental health disorders and NCDs: therefore, it is proposed as the fifth NCDs [5, 6]. Mental health disorders are the risk factors for CVD, cancers, and premature mortality [6]. Individuals with mental health disorders have a higher chance of premature death than that of the general population because of their physical health problems including suicide [7]. The suicide attempt is by far the most single strongest predictor of suicide in the general population [8]: it is the ultimate act of behavior that results from interactions of several factors including mental health disorders [9]. Globally, suicide is the second most common cause of death among young people [7]. Suicide cause 75% of death among young people in low- and middle-income (LMICs): about 90% of the world children and youths lives in LMICs [10]. One of the recognized strategies to prevent suicide is the assessment and management of individuals who attempted suicide [8].

Studies have shown that about 70% of premature death in adults is because of the lifestyle health-risk behaviors established during the adolescence period [11, 12]. About 36 million people die every year (63% of the global deaths) because of NCDs: fourteen million deaths are premature. LMICs contribute to 86% of the global NCDs premature deaths [1].

In sub-Saharan Africa, NCDs will be the leading cause of mortality by the year 2030 [12]. In Tanzania, all NCDs caused about 31% of premature deaths in the year 2012: the four main NCDs caused 16% of the deaths [13].

There is a limited number of studies about the co-occurrence and trends of risk factors for NCDs among in-school adolescents: most of the studies are from the middle- and high-income countries. For instance, two studies conducted in Brazil found that the co-occurrence of risk factors for NCDs was prevalent among in-school adolescents. In one of the study, it was found that adolescents had one (21.2%), two (37.3%), three (28.5%), and all (8.0%) risk factors for NCDs [14]. In the other study in-school adolescents were found to have the co-occurrence of two (22.2%), three (49.3%), four (21.7%), and five (3.1%) risk factors for NCDs. However, these risk factors were significantly more prevalent among older adolescents than younger adolescents [15]. In a Canadian study, the prevalence of risk factors for NCDs was found to co-occur in multiple combinations among in-school adolescents: however, higher among older adolescents than younger adolescents [16].

Schooling situation among adolescents in Tanzania was explored and reported by a study conducted in the year 2015 through 2016. The study revealed that about 3.5 million primary and secondary school-age children were out-of-school by the year 2012. However, by using the population projection from the 2012 Census data, in the year 2015 there were about 2.2 million and 1.7 million out-of-school children at the primary school level (aged 7—13) and at the secondary school level (aged 14—17), respectively. Moreover, the Demographic Health Survey and Malaria Indicator Survey (2015/16 TDHS-MIS) data reported that over 21.6% of primary-school-age children and 7.1% of secondary school-age children have never attended school [17].

Sub-Saharan Africa (SSA) is projected to have a large proportion and numbers of premature deaths attributed to NCDs by the year 2030 [12]. Currently, the burden of double disease in SSA is an indisputable reality, therefore, it is becoming urgent to address these risk factors for NCDs and more especially among adolescents. The NCDs related premature morbidity and mortality may jeopardize the ongoing achievement in tackling the major infectious diseases such as HIV/AIDs and tuberculosis in SSA. There is insufficient evidence about the prevalence and co-occurrence of risk factors for NCDs among adolescents in SSA. Moreover, it is unclear whether the sociodemographic presentation of risk factors for NCDs among adolescents in low-income countries is comparable to those in middle- and high-income countries. Therefore, the present study aimed at exploring the prevalence, sociodemographic distribution, and the co-occurrence of risk factors for NCDs among in-school adolescents in Tanzania as an example of a low-income setting of the SSA.

## Methods

### Data source

The present study is based on a secondary analysis of the first nationwide representative sample of the 2014 Tanzania Global School-based Student Health Survey (GSHS). The dataset used for analysis in the present study is free available in the following WHO repository website http://www.who.int/ncds/surveillance/gshs/tanzaniadataset/en/.

### Sampling technique and sample size

By adhering to the GSHS methodology, students were selected using a two-stage cluster sampling. The aim was to produce a nationwide representative sample of students from both primary schools (grades/standards 6–7) and secondary schools (form 1–3) aged 13–17 years. At the first stage of sampling, schools were selected with the probability proportional to their reported enrolment size in the national sampling frame. In total, 50 schools out of 20,230 schools were randomly selected to participate in the Tanzania GSHS. In the second stage, systematic random sampling was used to select classrooms from each selected school. All students in the selected classrooms were eligible to participate in the study regardless of their actual age. The school and student response rate was 100 and 87%, respectively. A total of 3,793 students participated and completed the study survey.

### Data management

The 2014 Tanzania GSHS, used a standardized questionnaire of the WHO GSHS to develop a country-based questionnaire. The questionnaire was translated into the Swahili language before pre-tested and used for the survey. Students who consented to participate in the survey were given a self-administered questionnaire. In order to protect the students’ privacy, the survey adhered to the principles of anonymous and voluntary participation. The survey used a weighing factor to each student to adjust for non-response and the varying probability of selection. The 2014 GSHS Tanzania was approved by the Tanzania Ministry of Health, Community Development, Gender, Elderly and Children (MoHCDGEC); and the Ministry of Education, Science and Technology (MoEST).

### Operational definitions of variables

*Tobacco use* means the use of any tobacco product in the past 30 days preceding the survey. The question was dichotomized as; the percentage of students who currently used any tobacco product (on at least 1 day during the 30 days before the survey). The variable response was dichotomized as “at least 1” day and “0 days”.

*Excessive alcohol consumption* was considered if the participant had ever drunk so much to the extent that he/she was really drunk at least once during his/her life. The following question was asked; During your life, how many times did you drink so much alcohol that you were really drunk? The variable response was dichotomized as “ drunk one or more” and “ never drunk ”.

*Unhealthy diet* was computed as a composite variable from the following four questions: (i) The proportion of students who did not eat fruit (during the 30 days before the survey), (ii) The proportion of students who did not eat vegetables (during the 30 days before the survey), (iii) The proportion of students who drunk carbonated soft drinks (excluding diet soft drinks) during the 30 days before the survey, and (iv) The proportion of students who ate food from a fast-food restaurant (during the 7 days before the survey). The response of each of the variable above was dichotomized as Yes and No. Then, a new composite variable *unhealthy diet* was computed from the four aforementioned variables. The composite variable response was then dichotomized as Yes (if yes to all the four variables), and No (if not to all or any combinations of no and yes).

P*hysical activity* was assessed according to WHO recommendations at least 60 min of exercise per day in at least 5 days per week for an adolescence population. Any kind of physical activity that could result in increased breathing and heart rate was considered. The following question was used; During the past 7 days, on how many days were you physically active for a total of at least 60 min per day? The variable response was dichotomized as “at least 5 days” and “less than 5 days” a week.

*Suicide attempt:* The question was dichotomized as: “During the past 12 months, how many times did you actually attempt suicide?” The variable response was dichotomized as “1,2,3,4,5, and ≥6 times attempts” and “ zero attempts”.

The other variables used in the present study were the participant’s demographics; age, gender, and school level (primary and secondary school).

### Data process and analysis

The prevalence of risk factors for NCDs among adolescents were examined and evaluated according to demographic characteristics; gender, age, and school level. The proportion of adolescents with one, and a combination of two, three, four and five, risk factors for NCDs were calculated and presented in percentage. The prevalence odds ratio was used to calculate one, and a combination of two, three, four and five, risk factors for NCDs, adjusted to gender, age, and school level.

Since nonresponses were observed to be missing at random (MAR) pattern, multiple imputation was used to examine the possibility of bias, however, no bias was observed. Replacing the missing data with imputed data did not make differences in outcomes of interest, hence a significant measure of association in both the bivariate and multivariate analyses was maintained despite imputation. A maximum of five imputations was run to allow for > 97% efficiency. A multiple imputation is a useful statistic technique replaces each missing value by two or more plausible values of nonresponse in public-use files. It is a useful statistic technique that replaces each missing value by two or more plausible values. The present study used the multiple imputation because of the following reasons; first, the pattern of missing data was missing at random [18–20], second, to account for the consistency of the dedicated sample size values of each variable throughout the analyses. Previous studies elsewhere have used a multiple imputation procedure to account for missing data at random [21–23].

The present study analysis used both descriptive and inferential statistics. Chi-square χ^2^ test, multinomial and multivariate regression models were used to determine the association between the variables of interest. In all analyses, the set level of statistical significance was a *p*-value of less than 0.05 at 95% confidence intervals. SPSS version 22 (SPSS, Chicago, IL) was used for the present analysis.

## Results

Table 1 represents the comparison of risk factors across the demographics of the study participants. Of the 3,793 in-school adolescents analyzed, 51.9% were female, and 78.8% were in the age group 13–17 years. Adolescents who were 12 years old and below had a significantly higher proportion of the unhealthy diet, tobacco use, excessive consumption of alcohol, and suicide attempt compared to other age groups.

**Table 1:** Risk factors for non-communicable disease according to demographics, *N* = 3,793

Variables	Total sample n (%)	Unhealthy diet n (%)	Physical inactivity n (%)	Tobacco use n (%)	Excessive alcohol n (%)	Suicide attempt n (%)
	3,793	3054 (80.5)	1044 (27.5)	311 (8.2)	138 (3.6)	427 (11.3)
Gender						
Male	1825 (48.1)	1445 (79.2)	443 (24.3)	159 (8.7)	75 (4.1)	194 (10.6)
Female	1968 (51.9)	1609 (81.8)	601 (30.5)	152 (7.7)	63 (3.2)	233 (11.8)
*P* value		0.004	< 0.001	0.29	0.14	0.26
Age (yrs)						
≤ 12	674 (17.8)	578 (85.8)	207 (30.7)	96 (14.2)	42 (6.2)	109 (16.2)
13–17	2988 (78.8)	2373 (79.4)	805 (26.9)	210 (7.0)	88 (2.9)	305 (10.2)
≥ 18	131 (3.4)	103 (78.6)	32 (24.4)	5 (3.8)	8 (6.1)	13 (9.9)
*P* value		0.001	0.09	< 0.001	< 0.001	< 0.001
School level						
Primary school	2085 (55.0)	1772 (85.0)	585 (28.1)	215 (10.3)	89 (4.3)	268 (12.9)
Secondary school	1708 (45.0)	1282 (75.1)	459 (26.9)	96 (5.6)	49 (2.9)	159 (9.3)
*P* value		< 0.001	0.58	< 0.001	0.03	0.001

### Prevalence of risk factors combination among study participants

The most prevalent combination of risk factors were unhealthy diet and physical inactivity 666 (17.6%); unhealthy diet and tobacco use 98 (2.8%); unhealthy diet and suicide attempt 151 (4.0%); and unhealthy diet, physical inactivity and suicide attempt 81 (2.1%).

### Association between the number of risk factors and demographics

Table 2 represents the multinomial regression of the association between a number of risk factors and adolescent’s demographics. In the adjusted model having three, and a sum of four and five risk factors than having no risk factor significantly declined with adolescent age. Male adolescents were significant less likely than females to have one 0.68 [0.55–0.84], two 0.64 [0.51–0.81], and a sum of four and five 0.61 [0.38–0.98] risk factors compared to having no risk factor.

**Table 2:** Multinomial regression of the number of risk factors and demographics, *N* = 3,793

Variables	Number of risk factors n (%)			
	One 2071 (54.6)	Two 968 (25.4)	Three 198 (5.2)	Four + five 89 (2.3)
	AOR [95% CI]			
^∫^Age (yrs)	1.10 [0.84–1.44]	0.92 [0.69–1.23]	0.60 [0.40–0.91] *	0.46 [0.28–0.79] **
Gender				
Male (Ref. Female)	0.68 [0.55–0.84] ***	0.64 [0.51–0.81] ***	0.81 [0.57–1.13]	0.61 [0.38–0.98] *
School level				
Primary school (Ref. Sec. school)	1.71 [1.37–2.14] ***	1.81 [1.42–2.32] ***	2.40 [1.63–3.54] ***	2.90 [1.61–5.13] ***

Adjusted for all independent variables.

*AOR* Adjusted odds ratio

Note: ^∫^Age is continuous, *** = *p* < 0.001, ** = *p* < 0.01 * = *p* < 0.05

### Association between the co-occurrence of risk factors and demographics

Table 3 represents the multivariate logistic regression of the combination of risk factors and adolescent’s demographics. In the adjusted model the increase of adolescents age was significant association with having the co-occurrence of the following risk factors; unhealthy diet and physical inactivity 1.24 [1.02–1.50]; unhealthy diet and suicide attempt 1.47 [1.13–1.90]; unhealthy diet and tobacco use 1.87 [1.41–2.49]; and unhealthy diet, tobacco use, alcohol use and suicide attempt 2.64 [1.48–4.78].

**Table 3:** Multivariate logistic regression of a prevalent combination of risk factors and demographics *N* = 3.793

Risk factors	Adjusted Odds ratio	Gender (Ref. Female) Male	^∫^Age (yrs)	School level (Ref. Sec. school) Primary school
U + P 666 (17.6)		1.19 [1.02–0.39]*	1.24 [1.02–1.50]*	0.89 [0.75–1.05]
U + S 151 (4.0)		1.11 [0.89–1.39]	1.47 [1.13–1.90]**	0.72 [0.56–0.92]**
	AOR (95% CI)			
U + T 98 (2.8)		0.84 [0.66–1.08]	1.87 [1.41–2.49]***	0.65 [0.48–0.87]**
U + P + S 81 (2.1)		1.03 [0.71–1.50]	1.35 [0.89–2.06]	0.55 [0.35–0.84]**
U + T + S 43 (1.1)		1.11 [0.85–1.45]	1.03 [0.74–1.42]	0.78 [0.58–1.05]
U + T + A + S 42 (1.1)		1.25 [0.73–2.13]	2.64 [1.48–4.78]**	0.71 [0.37–1.36]

Adjusted for all independent variables.

Note: U + P=Unhealthy diet + Physical inactivity, U + S = Unhealthy diet + Suicide attempt, U + T = Unhealthy diet + Tobacco use, U + P + S = Unhealthy diet + Physical inactivity + Suicide attempt, U + T + S = Unhealthy diet + Tobacco use + Suicide attempt, U + T + A + S = Unhealthy diet +Tobacco use + Excessive alcohol use + Suicide attempt

^∫^Age is continuous, *** = *p* < 0.001, ** = *p* < 0.01 * = *p* < 0.05

## Discussion

The present study finding showed that unhealthy diet constituted the highest proportion of all risk factors for NCDs followed by physical inactivity. Younger adolescents (≤12 yrs) than older adolescents (> 12 yrs) had a significantly higher proportion of the unhealthy diet, tobacco use, excessive alcohol consumption, and suicide attempt. Another finding was that the primary in-school adolescents than the secondary school adolescents had a significantly higher proportion of the unhealthy diet, tobacco use, and suicide attempt.

The present study finding is in contrast to the previous study conducted in Brazil. In Brazilian studies, the most prevalent risk factors were physical inactivity followed by the unhealthy diet: the proportion of excessive alcohol consumption was significantly higher in older adolescents than that in younger adolescents [14]. The common findings in both the Tanzania and Brazil studies were the high prevalence of the unhealthy diet and physical inactivity: this implies that the socio-cultural norms of the society may be unaware of the burden of these risk factors for NCDs. In SSA, the knowledge of risk factors for NCDs in particular unhealthy diet and physical inactivity among adolescents and youths has been reported to be poor [24]. On the other hand, risk factors such as tobacco use and excessive alcohol consumption may be considered by the society and policymakers to be less harmful to health: the tolerance of these risk factors in the society is uncertain. Therefore, the prevalence of tobacco use and excessive alcohol consumption may be under-reported than that of physical inactivity and unhealthy diet. Suicide attempt was the third most prevalent risk factor in the present study, however, this may be under-reported. This is because suicide behavior including suicide attempt is a sensitive issue in the society that is less disclosed or discussed in public: it is an illegal event in some countries across the world [8]. In the present study, the prevalence of risk factors for NCDs was higher in younger adolescents than that in older adolescents. There is a possibility of early experimentation of these lifestyle health-risk behaviors among adolescents in the Tanzania context.

The present study explored the co-occurrence of risk factors for NCDs among in-school adolescents in Tanzania as an example of the low-income country of sub-Saharan Africa. The most prevalent combination of risk factors for NCDs were two factors and three factors: the most prevalent co-occurrence factors were an unhealthy diet and physical inactivity; unhealthy diet and suicide attempt; and unhealthy diet and tobacco use. This finding is comparable to the previous studies conducted in middle-income countries [14, 15]: however, the proportion of these risk factors combination was relatively higher in middle-income countries than that in the present study. Moreover, in contrast to the present study, the previous studies focused on the independent prevalence of the risk factors for NCDs among adolescents [22, 25–29]. There is evidence that the co-occurrence of lifestyle, health-risk behaviours is more detrimental to health than that could be expected if the individual risk factors are added independently [30–37]. It has been suggested that multiple risk factors prevention approaches have a great impact at a lower cost than that of the individual risk factors prevention approach [38, 39]. Therefore, a multiple risk factors approach may be suitable for low-income and resources limited settings such as Tanzania: to mitigate the burden of NCDs risk factors among adolescence population.

In contrast to previous studies, the present study found that the co-occurrence of risk factors for NCDs significantly declined with adolescent age. Also, in the present study, the primary in-school adolescents were significantly more likely to have a combination of risk factors for NCDs than the secondary in-school adolescents. These findings suggest that younger adolescents in Tanzania are more likely to have multiple lifestyles, health-risk behaviours for NCDs than older adolescents. In LMICs, adolescents have a significantly high burden of lifestyle, health-risk behaviours for NCDs [40]: they are more affected by cultural, socio-economic, and environmental factors including early family responsibilities than their peers in high-income countries [41]. Therefore, the present finding suggests that NCDs preventive programs among adolescents in a low-income setting such as Tanzania may be started during early adolescence or to the adolescents in primary schools. The findings in middle- and high-income countries showed that older adolescents were significantly more likely than younger adolescents to have the co-occurrence of risk factors for NCDs [15, 16]. There is a disparity in the co-occurrence of lifestyle, health-risk behaviours for NCDs according to adolescents age and across the countries income status: this may save as an important definition criterion when deciding to implement NCDs prevention program.

Importantly, in contrast to previous studies, the present study included the suicide attempt as a risk factor for NCDs in the analysis. The study found that the suicide attempt was the third most prevalent risk factors, and it was also prevalent in combination with other NCDs risk factors. Suicide attempt is the most single strongest predictor of suicide in the general population [8]. Suicide cause 75% of death among young people in low- and middle-income (LMICs): about 90% of the world children and youths lives in LMICs [10]. Mental health disorders are the risk factors for CVD, cancers, and premature mortality [6]. There is an inextricable association between mental health disorders and NCDs [5]. It has been shown that adolescents with suicide behaviors such as suicide attempt are more likely to have other NCDs risk factors such as physical inactivity, tobacco use, and alcohol consumption [42, 43].

The present study has several strengths. First, this may be the first study in a low-income setting of SSA to have explored the co-occurrence of risk factors for NCDs among in-school adolescents. Most of the previous studies were from middle-and high-income countries. Therefore, the present study finding in Tanzania may save as an example of a low-income setting of sub-Saharan Africa. Second, in contrast to the previous studies in middle- and high-income countries, the present study showed that the co-occurrence of risk factors was significantly prevalent among younger adolescents. Third, the present study analysed the suicide attempt variable: previous studies did not consider to analyse the variable. Suicide attempt was prevalent both as an independent and in co-occurrence with other risk factors for NCDs. Fourth, the present study used multiple imputation to examine the effect of missing data, which is common in survey studies.

The standard GSHS questionnaire is a fast, cost-efficient, easy-to-use tool for assessing the adolescent’s health behaviours in risky and resources-constrained populations. However, it has several limitations and especially when used in the Tanzania context. First, the GSHS involved adolescents who were attending school and therefore the finding may not be generalizable to the out-of-school adolescents. In the year 2014, the gross enrollment of pupils in Tanzanian primary and secondary school was 82.62 and 31.67%, respectively [44]. There were about 2.2 million out-of-school children at the primary school level (aged 7—13), and 1.7 million out-of-school children at the lower secondary school level (aged 14—17) by 2015 [17]. Second, since the data was self-reported, it relied upon study participants recall memory, therefore, there could be potential recall bias. Third, the present study could not measure the chronicity of the risk factors for NCDs because of its cross-sectional design nature. This limits its utility to explain the temporal association of risk factors and adolescent’s demographics. Although the results give a significant insight about the co-occurrence of the risk factor for NCDs among adolescents, we should interpret the results with caution when comparing with findings from other types of study design. Furthermore, the present study did not measure a chemical biomarker to ascertain the use of tobacco or alcohol.

## Conclusions

The present study showed clear evidence that the co-occurrence of risk factors for NCDs exists and prevalent among adolescents in Tanzania. However, in the present study, younger adolescents were significantly more likely to have a higher prevalence and co-occurrence of risk factors for NCDs. A multi-strategy public health intervention study may be of cost-effective than a single risk factor approach hence suitable for resource-limited settings, such as Tanzania.

## Data Availability

The datasets file used for analysis in the present study is publicly available for free in the WHO repository (http://www.who.int/ncds/surveillance/gshs/tanzaniadataset/en/).

## Abbreviations

AIDS: Acquired Immune Deficiency Syndrome
AOR: Adjusted odds ratio
CVD: Cardiovascular Disease
GSHS: Global School-based Student Health Survey
HIV: Human Immunodeficiency Virus
LMICs: Low and Middle-income countries
MoEVT: Ministry of Education and Vocational Training
MoHSW: Ministry of Health and Social Welfare
NCDs: Non-communicable Diseases
NSHP: National School Health Programme
OR: Odds ratio
WHO: World Health Organization
